# Unmet needs in the management of immune‐mediated thrombotic thrombocytopenic purpura and the potential role of caplacizumab in the UK—A modified‐Delphi study

**DOI:** 10.1002/jha2.435

**Published:** 2022-04-28

**Authors:** Marie Scully, Tina Dutt, Will Lester, Emily Farrington, Stevie Lockwood, Richard Perry, Steve Holmes

**Affiliations:** ^1^ Department of Haematology University College London Hospital London UK; ^2^ Liverpool University Hospitals, NHS Foundation Trust Liverpool UK; ^3^ Centre for Clinical Haematology NHS Foundation Trust University Hospitals Birmingham Birmingham UK; ^4^ Adelphi Values UK Bollington UK; ^5^ Sanofi Paris France

**Keywords:** ADAMTS13, caplacizumab, management, thrombocytopenic purpura, unmet need

## Abstract

Immune‐mediated thrombotic thrombocytopenic purpura (iTTP) is an ultra‐rare, blood‐clotting disorder. Management historically relies on plasma exchange and immunosuppression; however, a 10%–20% mortality rate is still observed. Caplacizumab binds to von Willebrand factor and directly inhibits platelet aggregation; addition of caplacizumab to historical treatment induced faster resolution of platelet count in clinical trials. In 2019, a modified‐Delphi study was conducted with UK experts, to develop consensus statements on management of acute TTP and the potential role of caplacizumab. An unmet need was acknowledged, and areas requiring improvement included: time to diagnosis and treatment initiation; time to platelet normalisation (TTPN) during which patients remain at risk of persistent microvascular thrombosis and organ damage; and incidence of subsequent exacerbations and relapses. Caplacizumab addition to historical treatment within 24 h (after confirmatory ADAMTS13 [a disintegrin and metalloproteinase with thrombospondin type 1 motif, member 13] assay) would significantly reduce TTPN, which directly influences acute outcomes, with manageable bleeding risk and reduced burden on healthcare systems. Expert panellists agree that poor outcomes in iTTP largely result from failure to rapidly control microvascular thrombosis. Use of caplacizumab during a confirmed iTTP episode could offer better control and may plausibly improve long‐term outcomes. However, this consensus must be validated with further clinical trials and robust real‐world evidence.

## 1 INTRODUCTION

Immune‐mediated thrombotic thrombocytopenic purpura (iTTP), also known as acquired TTP (aTTP), is due to a deficiency in the activity of ADAMTS13 (a disintegrin and metalloproteinase with thrombospondin type 1 motif, member 13), caused by an autoantibody to the enzyme. iTTP is a life‐threatening and extremely rare blood‐clotting disorder, with an approximate incidence of 600 suspected cases in the UK in the last 10 years [1]. National Health Service (NHS) England estimate 100–150 acute admissions for TTP per year in England [2]. The rarity of the condition and its typically sudden onset make iTTP particularly challenging to manage, which can lead to fatal outcomes [3, 4].

The pathological mechanism of iTTP is a severe deficiency in the activity of the von Willebrand factor (VWF) cleaving enzyme, ADAMTS13 [5, 6]. Autoantibodies bind to ADAMTS13, increasing its clearance and inhibiting its function, allowing VWF to circulate uncleaved, leading to the persistence of ultra‐large von Willebrand factor (ULVWF) multimers in plasma [3]. ULVWF multimers are extremely adhesive to platelets in the circulation, resulting in the spontaneous formation of microvascular thrombi [5, 7–9]. This leads to partial vessel occlusion, which can ultimately result in microangiopathic haemolytic anaemia (MAHA), organ ischaemia and widespread organ damage [10].

The risk of mortality associated with iTTP makes it crucial that clinicians act rapidly. Patients who newly present with the disease are treated as a medical emergency as without intervention the survival rate is less than 10% [4, 7, 10–12]. However, diagnosis is challenging as patients can present with non‐specific clinical symptoms [4]. Tissue ischaemia and organ dysfunction often involves the brain, heart and kidneys, and therefore the immediate concern is to prevent severe acute consequences associated with myocardial and cerebral damage [13, 14, 15]. The rarity of the condition and limited follow‐up data means the specific long‐term outcomes remain poorly defined, but survivors of acute episodes often describe problems associated with cognitive impairment, such as difficulty with memory, concentration and endurance [16, 17]. Furthermore, survivors are at lifetime risk of relapse, with each acute episode carrying similar risk of organ damage, mortality and morbidity [7].

**What is the new aspect of the work?**

This research establishes a clinical consensus on modern treatment pathways for immune‐mediated thrombotic thrombocytopenic purpura (iTTP), an ultra‐rare condition.

**What is the central finding of the work?**

An unmet need exists in iTTP in terms of time to diagnosis and treatment initiation (and subsequent outcomes, including relapse risk); addition of caplacizumab to historical treatment options would significantly reduce time to platelet normalisation, and therefore improve acute outcomes in iTTP.

**What is the specific clinical relevance of the work?**

The rarity of iTTP can translate to limited clinical experience; however, this modified‐Delphi study provides a clinical consensus on iTTP management from consultant haematologists and pharmacists in the UK (*N* = 10), according to a robust qualitative and quantitative methodology.

The primary treatment goal of acute TTP is inducing remission, defined as a normal platelet count, through control of microvascular thrombosis [10]. Historical treatment consists of plasma exchange (PEX) and immunosuppression, which aims to remove ULVWF and autoantibodies, replace functional ADAMTS13, and prevent further formation of autoantibodies [3]. Caplacizumab, a humanised Nanobody (Nanobody is a trademark of Ablynx, a Sanofi company) has recently been evaluated in combination with historical treatment in a Phase III study, and is now reimbursed by the UK NHS following health technology assessment in England and Scotland [18]. Caplacizumab targets the A1 domain of VWF and inhibits binding to platelets, thus rapidly inhibiting platelet aggregation to prevent the formation of microthrombi and subsequent ischaemic organ damage [19, 20].

While clinical studies and guidelines for iTTP management exist, the rarity of iTTP can translate to limited clinical experience. Evidence generation in rare diseases often requires use of alternative methods, including expert consensus, to gain a fully comprehensive overview of the disease. Therefore, we present the results of a modified‐Delphi panel with UK experts in iTTP, which verifies and adds to the existing literature. The specific aims were to develop a deeper understanding of the current management and unmet need for patients experiencing an iTTP episode, while considering the potential role for caplacizumab.

## 2 METHODOLOGY

**FIGURE 1:**
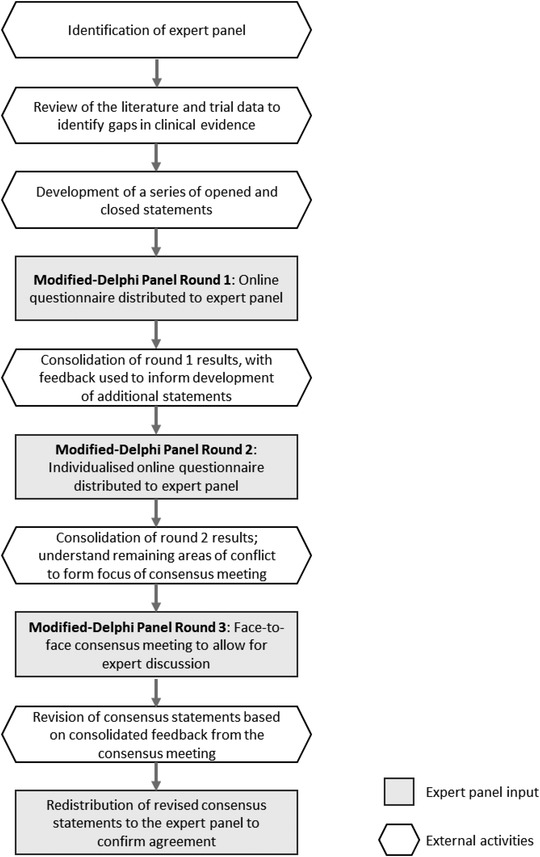
Modified‐Delphi panel process

A three‐step modified‐Delphi panel was conducted between February and March 2019, which incorporated two rounds of anonymised online surveys and a face‐to‐face consensus meeting (Figure 1). The process was designed to understand panel perceptions on the unmet need in patients experiencing an acute iTTP episode in terms of diagnosis, current treatment strategies and the impact upon health consequences, and to define the potential role of caplacizumab. Although this condition was referred to as aTTP during the panel, the term iTTP is used throughout this report to reflect that iTTP is now the more frequently used terminology.

The first survey round comprised closed statements and open questions, based on data available in the literature or clinical trials. Panellists were asked to rate all closed statements and were encouraged to provide rationale for their answers, and any additional comments. The second round clarified areas nearing consensus and identified aspects that required discussion in the consensus meeting. The results from the second‐round survey were consolidated, analysed and presented at the consensus meeting.

Both survey rounds took 30–60 min to complete online survey and panellists were given 2 weeks to complete each one. The iterative nature of the process meant surveys were adapted between rounds based on panellist feedback; statements that reached consensus and areas deemed illogical to take forward were excluded. Statements that did not reach consensus were modified based on qualitative responses.

**FIGURE 2:**
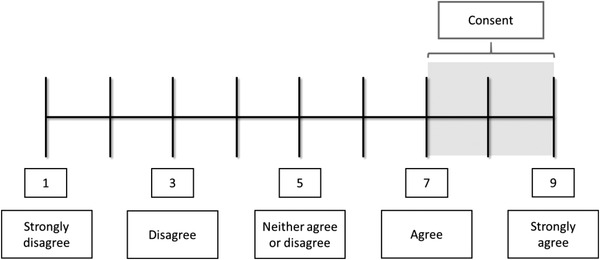
The nine‐point Likert scale used to determine consensus

For closed statements, panellists were asked to rate their agreement based upon a nine‐point Likert scale; with 1 considered 'completely disagree' and 9 considered 'completely agree'. Consensus was defined as: ≥80% of panellists rated their ‘agreement’ between 7 and 9 (Figure 2).

The objective of the face‐to‐face meeting was to gain agreement on the remaining areas of conflict and to confirm the areas of consensus. The panel was provided with a brief overview of statements that had reached consensus and the Delphi round results for the areas of conflict. During the meeting, statements could be adapted based on consolidated feedback and the panel were asked to rate their agreement with the modified statements at numerous checkpoints.

As this was a modified‐Delphi process, it was critical that measures were taken to minimise bias. Anonymity was maintained throughout the first two Delphi rounds to ensure initial perspectives were not influenced by other panellists. The consensus meeting and discussions were facilitated by a third‐party to ensure all opinions were heard and the sponsor company was not present.

## 3 RESULTS

### 3.1 Expert panel

Eleven experts comprising consultant haematologists (*N* = 9) and pharmacists (*N* = 2) across the UK (England: *N* = 10, Scotland: *N* = 1), agreed to participate and were recruited. One pharmacist withdrew from the process after the first round, and thus 10 panellists completed the process (England: *N* = 9, Scotland: *N* = 1).

### 3.2 Overview of consensus

Following the consensus meeting, the statements were revised to achieve 14 final consensus statements. While the definition for consensus, as described in the methods, was used throughout the study, 100% (*N* = 10) of the final panel agreed to all finalised statements—therefore ratings of consensus are not provided.

### 3.3 Diagnosis

**FIGURE 3:**
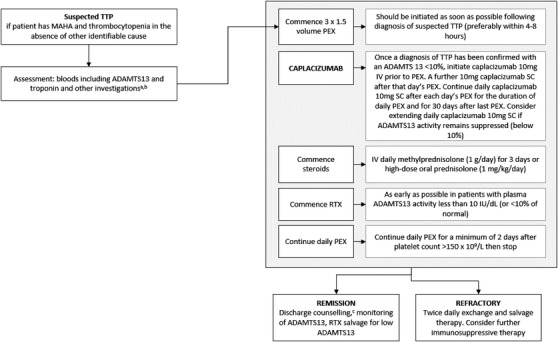
The diagnostic and treatment pathway for immune‐mediated thrombotic thrombocytopenic purpura (iTTP). (A) Take blood before starting plasma exchange (PEX): full blood count (FBC), blood film reticulocytes, clotting, fibrinogen, urea and creatinine, troponin I/troponin T, liver function tests (LFTs), amylase, thyroid function tests (TFTs), calcium, lactate dehydrogenase (LDH), pregnancy test, direct antiglobulin test, blood pressure, blood group with antibody screen, ADAMTS13 (a disintegrin and metalloproteinase with thrombospondin type 1 motif, member 13), hepatitis A/B/C, human immunodeficiency virus (HIV) serology and autoantibody screen. (B) Other investigations should be performed promptly but can be delayed until after starting PEX: urinalysis, stool culture (if diarrhoea), echocardiogram, computerised tomography (CT) brain (if neurological signs), and CT chest/abdomen/pelvis to check for underlying malignancy (if indicated). (C) Patients should be counselled about symptoms, signs and risk of relapse before discharge with verbal and written information. Abbreviations: IV, intravenous; MAHA, microangiopathic haemolytic anaemia; RTX, rituximab; SC, subcutaneous; TTP, thrombotic thrombocytopenic purpura. Adapted from Scully et al. [10]; rituximab recommendations adapted from Dutt et al. [29] and Zheng et al. [32]

Diagnosis of iTTP can be challenging given the complex and variable clinical presentations, which can be indicative of other conditions [7, 10]. iTTP is characterised by thrombocytopaenia, MAHA and the formation of microthrombi [10, 11], but patients may also present with fluctuating neurological signs, renal impairment and fever depending on the individual case [10]. Diagnostic criteria state that when patients present with thrombocytopaenia and MAHA alone, with no other identifiable clinical cause, iTTP must be considered [10, 11]. As ADAMTS13 activity is crucial to the disease mechanism, it is important that an ADAMTS13 assay is conducted to assess baseline ADAMTS13 activity; severely reduced activity can be used to confirm iTTP diagnosis and monitor the disease (Figure 3) [10].

#### 3.3.1 Expert consensus

1. If a patient presented with severe thrombocytopaenia and MAHA, I would diagnose probable iTTP, initiate PEX and aim to initiate caplacizumab within 24 h subject to receiving a confirmatory ADAMTS13 assay.

ADAMTS13 assay result of <10% confirms a diagnosis of TTP. This would be expected to be processed within 24 h.

2. Lower platelet count is often related to greater disease severity (which can also be indicated by the biochemical marker troponin and neurological symptoms), but this is subject to the variable clinical situation.
3. The difficulty and time taken for diagnosis and initiation of treatment contribute to an unmet treatment need.

### 3.4 Current treatment

Historical treatment for iTTP involves a combination of PEX and immunosuppression [3]. PEX is the most important acute intervention and works by replenishing functional ADAMTS13 while also removing the ADAMTS13 autoantibodies and ULVWF multimers [3]. Immunosuppression with corticosteroids inhibits further autoantibody formation and has demonstrated improved patient outcomes (e.g. reductions in the number of PEX required to achieve remission, the length of inpatient stays and relapse rates) [3, 20–22]. With prompt initiation of PEX and immunosuppression, the survival rate improves substantially to 80%–90%, compared to less than 10% with no treatment [7]. However, despite the greatly improved outlook for patients, the remaining mortality rate and other associated health consequences indicate a need for treatment improvement [23].

#### 3.4.1 Expert consensus

1. PEX and immunosuppression are routine management for iTTP and reduce the risk of mortality and severe organ damage (vs. no treatment) but may not eliminate long‐term consequences.
2. Following PEX and immunosuppression patients may experience exacerbations and/or relapses* and a subset of patients do not respond, all of which contribute to an unmet treatment need.

Exacerbation: defined as a reduction in platelet count to below the lower limit of the established reference range (e.g. <150 × 10^9^ L^−1^), an increased lactate dehydrogenase level, and the need to restart PEX within 30 days of the last PEX after a clinical response to PEX [24].

Relapse: defined as a fall in platelet count to below the lower limit of the established reference range (e.g. <150 × 10^9^ L^−1^), with or without clinical symptoms, >30 days after stopping of PEX for an acute TTP episode, requiring re‐initiation of therapy [24].

### 3.5 Current treatment impact: Acute and long‐term health consequences

Acutely, iTTP patients are at risk of cardiac and cerebral tissue ischaemia, and potentially catastrophic thromboembolic events such as myocardial infarction or stroke [25, 26]. The longer it takes for a treatment response, the longer a patient remains at risk of microthrombotic complications, tissue ischaemia and potential organ damage [20]. There is limited clinical evidence that evaluates long‐term health consequences, but patients that recover from an acute episode often describe problems associated with neurocognitive impairment and neuropsychological symptoms [17]. While PEX and immunosuppression greatly improves mortality rates for iTTP patients, it is also important to understand the impact on other acute and long‐term health consequences.

#### 3.5.1 Expert consensus

There is an unmet treatment need in iTTP, mostly associated with a failure to achieve rapid control of microvascular thrombosis.

1. The time taken for platelet normalisation with PEX and immunosuppression results in patients remaining at risk of persistent microvascular thrombi and organ damage during the acute phase of iTTP.
2. The longer a low platelet count persists, the longer the patient remains at risk of acute and long‐term complications as a consequence of microvascular thrombosis.
3. Some iTTP survivors following PEX and immunosuppression are left with long‐term health consequences—likely due to delay in diagnosis and failure to achieve normalisation of platelet count.

### 3.6 Potential benefits of a novel therapy, caplacizumab

Two randomised placebo‐controlled trials, TITAN (Phase 2) and HERCULES (Phase 3), investigated the effect of adding caplacizumab to PEX and immunosuppression during an acute iTTP episode [18, 20]. These studies demonstrated that caplacizumab, in combination with PEX and immunosuppression, rapidly inhibits microvascular thrombosis, significantly reducing the time to platelet count normalisation (TTPN) versus PEX and immunosuppression alone [18, 20]. Through rapidly preventing microthrombi formation caplacizumab prevents further ischaemia‐induced organ injury [20], but the potential long‐term benefits are yet to be evaluated. Therefore, it was important to gather panel insights on how caplacizumab's mechanism of action may impact patients in the short and long term.

#### 3.6.1 Expert consensus

1. TTPN is directly related to the acute outcomes and it is biologically plausible that it is directly related to long‐term consequences.
2. Caplacizumab plus PEX and immunosuppression significantly reduces TTPN compared to PEX and immunosuppression alone, reducing the risk of acute ischaemic events. Through a reduction in the thrombotic burden associated with an acute episode of TTP, this has the potential to prevent some of the long‐term consequences of such episodes, although this requires further study and follow‐up.

TITAN indicated that caplacizumab, through a faster platelet count normalisation, prevents further platelet aggregation into microthrombi and the consequent tissue ischaemia [20]. Post hoc analysis of the TITAN study found that fewer caplacizumab‐treated patients had a major thromboembolic event, an exacerbation or died versus placebo [27]. Based on this the authors concluded that caplacizumab has the potential to reduce the acute morbidity and mortality associated with iTTP [27]. Following this, the HERCULES trial demonstrated that during the treatment period, caplacizumab was associated with a more rapid normalisation of platelet count than PEX and immunosuppression alone, and a lower incidence of a composite of TTP‐related death, major thromboembolic events, and recurrence of iTTP [18]. In addition, there were no patients in the caplacizumab arm of the HERCULES trial with refractory disease, compared to three patients in the placebo group [18]. Furthermore, treatment with caplacizumab reduced the mean number of PEX days and plasma volume administered compared to the placebo group, which ultimately resulted in a shorter hospital stay [18, 20]. Based on the available evidence and experts’ experience, further understanding of the role caplacizumab could play in the management of iTTP was sought.

#### 3.6.2 Expert consensus

1. Based on its mode of action, caplacizumab plus PEX and immunosuppression (compared to PEX and immunosuppression without caplacizumab) would substantially reduce the relative risk of:
  i. Mortality
  ii. Organ damage
  iii. Myocardial ischaemia
  iv. Cerebral ischaemia
  v. Length of ICU/hospital stay
  vi. PEX
  vii. Exacerbations
2. Based on its mode of action, it is biologically plausible that caplacizumab plus PEX and immunosuppression (compared to PEX and immunosuppression without caplacizumab) would reduce the risk of long‐term consequences associated with acute organ damage, such as neurocognitive complications, which are prevalent in this population.
3. Caplacizumab plus PEX and immunosuppression would improve the management of an iTTP episode, reducing the burden on the healthcare system (length of stay, ICU days, use of PEX, early re‐admission).

On the basis of caplacizumab's pharmacological effect, interfering with VWF binding to platelets, it can be associated with mucocutaneous bleeding [18]. During the randomised controlled trials, bleeding events were observed more frequently in the caplacizumab treatment group compared to placebo, but these were generally mild and did not require intervention [18, 20]. Therefore, it was important to further understand expert perceptions on the tolerability of caplacizumab.

#### 3.6.3 Expert consensus

The bleeding risk associated with caplacizumab is usually minor and generally manageable; however, drug costs and the need for national guidelines are potential barriers for caplacizumab use.

## 4 DISCUSSION

iTTP is challenging to diagnose and demanding to treat, and the rarity of the condition means evidence on the disease must also be gathered in routes outside of traditional clinical studies. There are limited numbers of healthcare practitioners with extensive experience managing iTTP, making it crucial to consolidate and communicate their clinical knowledge. An unmet need for iTTP patients was acknowledged, and the areas requiring improvement to achieve the best possible care were indicated. Overall, the consensus statements supported the information available in the literature and clinical trials, but also added to the current knowledge base regarding the expected impact caplacizumab may have on iTTP management.

During this modified‐Delphi process, certain aspects, such as the diagnosis and unmet need in iTTP, reached consensus quickly, but areas where further discussion was needed highlighted the need to generate additional clinical evidence. Areas relating to the long‐term outcomes associated with iTTP were discussed in detail. The panellists acknowledged that until remission is achieved, patients remain at risk of microthrombotic complications and tissue ischaemia, potentially leading to long‐term and irreversible health consequences. Neurocognitive complications are prevalent in iTTP survivors. Anxiety and depression have also been reported as potential long‐term outcomes—these may be linked directly or be the consequence of survivors living with a chronic, life‐threatening condition, susceptible to relapse. Ultimately, more data are required to reliably define the specific outcomes associated with iTTP and to evaluate the long‐term impact of treatment with caplacizumab in patients with iTTP.

All patients who suffer from a life‐threatening condition deserve to receive the best possible care, but this is often challenging for rare conditions where there is less clinical knowledge and fewer treatment options [4]. Caplacizumab is the first treatment specifically developed to treat iTTP, thus it was important to gather expert insights on how the availability of this therapy may alter the treatment landscape. Overall, the expert panel expressed that use of caplacizumab during a confirmed iTTP episode would offer better control of microvascular thrombosis and potentially improve the long‐term outlook for patients. However, it was acknowledged that these opinions are hypothesis‐generating and need to be validated with further clinical trials and robust real‐world evidence.

Long‐term evidence, incorporating follow‐up of iTTP survivors and development of prospective registries, is required to accurately understand the long‐term health outcomes associated with ongoing tissue ischaemia during an acute iTTP episode. Extended clinical studies to evaluate the long‐term benefits of caplacizumab will provide a more comprehensive picture—a 3‐year follow‐up study is underway for patients who completed the HERCULES trial with an aim to characterise the long‐term impact of iTTP and caplacizumab treatment [28].

Since the conduct of this modified‐Delphi study, further evidence on caplacizumab has been published [29, 30, 31]. A 2021 retrospective study of 85 patients receiving caplacizumab in UK hospitals demonstrated that use of this treatment in the real world is associated with comparable outcomes to the trial setting in terms of parameters such as TTPN and length of PEX, both of which were superior to historical treatment alone (*p* < 0.05) [29]. In addition, in four of five fatal cases, caplacizumab was introduced more than 48 h after initiation of historical treatment [29]. A 2021 study of 90 caplacizumab‐treated patients in France reinforced these findings, showing that the combination of caplacizumab, PEX and rituximab as front‐line therapies led to a significantly reduced rate of unresolved disease or death by Day 30, a significantly reduced rate of exacerbation, and significantly reduced TTPN (all *p* < 0.01), versus a historical control group [30]. A further 2020 retrospective study (without a comparative element) of 60 patients receiving caplacizumab in Germany reported that TTPN occurred within a median of 3 days; in one fatal case, caplacizumab initiation was late [31].

Of note, in December 2020, caplacizumab was recommended by the UK National Institute for Health and Care Excellence (NICE) as an option for the treatment of aTTP, in combination with PEX and immunosuppression. The conclusions of this project formed part of the dossier submitted for review by NICE.

## 5 LIMITATIONS

Although this modified‐Delphi process was conducted in a robust manner, limitations were apparent. A modified approach utilising a consensus meeting was deemed beneficial for obtaining expert insights, but this removed anonymity. A lack of anonymity could inadvertently lead to the suppression of less popular views among the panellists; however, every effort was made to encourage balanced participation from all panellists and consensus was repeatedly sought throughout and following the meeting. Additionally, as the panel were aware this was a sponsored study, a level of bias might be expected. However, the sponsor was excluded from all discussions to minimise this potential bias.

## 6 CONCLUSIONS

The consensus statements presented reflect the clinical opinion of experts in the management of iTTP across England and Scotland. Historical treatment of iTTP with PEX and immunosuppression has demonstrable effect and improves mortality rates, but there remains an unmet treatment need for patients with iTTP. This unmet need comprises difficulties with diagnosis and immediate initiation of treatment, failure to achieve rapid control of microvascular thrombosis, and the potential for no response to treatment or disease exacerbations after an initial response. These complications can lead to fatal consequences acutely, and potential long‐term morbidity, such as neurological impairment, for those who survive. In the opinion of the panel, there is potential for caplacizumab, in combination with PEX and immunosuppression, to improve the lives of patients and reduce the burden on the healthcare system, through reducing the risk of detrimental health outcomes compared to PEX and immunosuppression alone. Early data from national cohorts published in 2020 and 2021 indicate a positive effect of the more widespread use of caplacizumab in the management of acute iTTP; however, robust follow‐up will be crucial to better understand the long‐term impact of caplacizumab.

## PATIENT CONSENT STATEMENT

Clinical consensus statements have been provided for informational purposes. The clinical consensus statements are based on the opinions of a UK panel, but do not represent clinical practice guidelines. Consideration of clinical consensus statements will not ensure successful patient outcomes in every situation, and ultimately the treatment, diagnosis and management must be determined by the responsible HCP.

## CONFLICT OF INTEREST

The authors declare they have no conflicts of interest.

## AUTHOR CONTRIBUTIONS

Marie Scully, Tina Dutt and Will Lester formed the steering committee of the expert panel and critically reviewed and revised the manuscript. Emily Farrington, Stevie Lockwood and Richard Perry designed the project alongside Steve Holmes, conducted all stages, analysed the results and drafted the manuscript. All authors reviewed and approved the final manuscript.

## Data Availability

The data analysed and generated within this study are available from the authors upon reasonable request.
